# From Recruitment to Retirement: Research Infrastructure Staff Views on the Diversification of Scientific Career Paths at Universities in Sweden in 2024

**DOI:** 10.12688/f1000research.164794.1

**Published:** 2025-07-04

**Authors:** Kristen Schroeder, Julia Fernández-Rodríguez, Annika Jenmalm-Jensen, Josefin Lundgren-Gawell, Sara Sandin, Charlotte Stadler, Jessica M. Lindvall

**Affiliations:** 1 Science for Life Laboratory, Training Hub, Department of Biochemistry and Biophysics, Stockholm University, Stockholm, Sweden; 2 Science for Life Laboratory, Centre for Cellular Imaging, University of Gothenburg, Gothenburg, Sweden; 3 Science for Life Laboratory, Department of Medical Biochemistry and Biophysics, Karolinska Institute, Science for Life Laboratory, Huddinge, Sweden; 4 Science for Life Laboratory, KTH - Royal Insitute of Technology, Stockholm, Sweden; 5 Science for Life Laboratory, Department of Chemistry, Umeå Centre for Electron Microscopy, Umeå University, Umeå, Umea, Sweden; 6 Science for Life Laboratory, Department of Protein Sciences, KTH - Royal Institute of Technology; Science for Life Laboratory, Stockholm, Sweden

**Keywords:** research infrastructure, career paths, science and society, Sweden, life science

## Abstract

**Background:**

There is an ongoing need to develop diverse career paths that support the vital contributions of staff scientists, research engineers, scientific officers, and other knowledge professionals in scientific discovery. Research infrastructures and core facilities have a particular need to support sustainable and diverse careers, as they either employ – if being a legal entity – or daily manage research professionals in a broad variety of roles to enable resources, services, and innovation.

**Methods:**

In 2019, a survey of the facility staff at SciLifeLab, a large national research infrastructure in Sweden, led to a recommendation for universities to develop career paths for their staff scientists. Five years later, we have conducted a survey and workshop to determine current views of infrastructure staff on career path diversity in Sweden.

**Results:**

Our results indicate there is a strong need for clarity and communication about planning and implementation of career path structures at Swedish universities, as well as opportunities to foster excellence in infrastructure staff. While the workshop participants ranked Sweden as a stable and attractive place to work and reported continuous development of their technical and service skills, the lack of recognition of this expertise presents a barrier to a sustainable career.

**Conclusions:**

We conclude that there is a need to continue advocating for increased clarity and diversity in career paths for staff scientists in Sweden, and raise the views presented by infrastructure staff on the challenges and opportunities unique to their roles.

## Introduction

The structure of scientific organizations has diversified from individual professors leading a team of trainees to teams of research scientists running larger infrastructure facilities and centers. ^1, 2^ However, the career path from graduating with a university degree to retirement has not kept pace with the growing number of experts working outside the traditional academic career track. Large national research infrastructures (RIs) and Core Facilities (CF) now unite thousands of experts including staff scientists, technical specialists, and administrative and organizational support personnel. Within these, there is a recognized need to increase visibility and support sustainable career development for these knowledge professionals. ^2– 4^ In countries such as the United Kingdom, ^5^ Belgium, ^1^ and the Netherlands, ^6^ as well as in field-specific initiatives, ^7^ surveys and assessments have been conducted to better understand specific challenges for scientists and experts outside the traditional academic track. These analyses have consistently highlighted the need for greater diversity in career path structures within core facilities, ^4^ with ongoing efforts focused on organizing and defining tailored career paths for individual communities. ^1, 7^

In Sweden the research landscape consists of over 60 nationally distributed RIs, one being SciLifeLab ( https://www.scilifelab.se/) which brings together approximately 600 dedicated infrastructure staff—employed through all major Swedish universities—across various expert and knowledge professional roles. SciLifeLab operates ten technology platforms and more than 40 facility units within data- and technology-driven life science, with additional support infrastructure focused on data services and management, training, and operations. With a high percentage of employees in roles outside of the traditional academic career track, SciLifeLab is committed to promoting diversity in research careers and is a signatory of CoARA, ^8^ a coalition of institutions dedicated to reforming the evaluation of research contributions.

In 2020, the Association of Swedish Higher Education Institutions (Sveriges Universitet och Högskoleförbund, SUHF) established a Swedish reference group (Universitetens Referensgrupp för Forskningsinfrastruktur, URFI) to determine staff categories with central roles and ask for views on career progression opportunities and obstacles from the staff within these categories. As part of the report from URFI, a survey was conducted across two of the governmental RIs in Sweden; SciLifeLab and MAX IV. The results of this initial survey on career paths for staff scientists were presented at the 2020 SciLifeLab Facility Forum, ^9^ a meeting gathering infrastructure staff across SciLifeLab units. Discussions during this session highlighted the need for clearer and more distinct career path structures for staff scientists in Sweden, separate from the traditional academic research or teaching tracks. Later that year, the first report on career paths for staff scientists in Sweden was published, ^9^ and in 2023 the Karolinska Institute approved new roles for technical staff. ^10^ Today, implementation of the URFI recommendation of a two-stage career path is ongoing at several universities in Sweden, with support for diversified career paths from agencies such as the Swedish Research Council. ^11^

Building on the previous work and survey outcome, we organized the workshop “From Recruitment to Retirement” for the 2024 SciLifeLab Facility Forum, to ask infrastructure staff for their current views on how scientific career path diversity has developed over the past four years in Sweden. In this article, we report the findings from this workshop.

## Methods

### Participants

The participants in this study included research infrastructure staff working at SciLifeLab with varied levels of university training and a broad range of titles. Two thirds of SciLifeLab infrastructure staff hold a PhD, and job titles for these positions include data stewards, lab managers, systems developers, research engineers in technology design and technology services, scientific officers, bioinformaticians, team leads, administration and coordination roles.

### Survey and workshop structure

The workshop was carried out at the 2024 SciLifeLab Facility Forum, a semi-annual research infrastructure meeting that invites all employees across all technology platforms, plus operational, data management, and training units. Workshop sign-up was available to all Facility Forum participants, with a maximum of 65 seats, and was designed to consist of three components; 1) a survey conducted prior to the workshop, 2) the workshop presentation, and 3) real-time feedback from the workshop participants. Participants were advised before proceeding with the survey that all responses collected would be anonymized and only be presented in aggregate if there were enough responses to reasonably assure anonymity. In submitting the survey, participants gave informed consent to their responses being presented in this way, and during the workshop participants were informed that the collected responses would be published after the workshop. Participation in all aspects of this study was voluntary.

To tailor the workshop to the perspective of those attending, the pre-workshop survey was distributed beforehand by email to all registered participants, and a response rate of 25/65, or 40% was achieved. After receiving responses, free text entries were coded to remove identifying information and shown in aggregate as part of the workshop presentation. During the in-person workshop, further questions were posed to the larger group of participants with the assistance of Mentimeter (Mentimeter AB, Sweden), followed by smaller group discussions. Realtime responses were coded to remove identifying information and are presented alongside the survey results here.

## Results

### Pre-workshop survey and presentation

**Figure 1.**
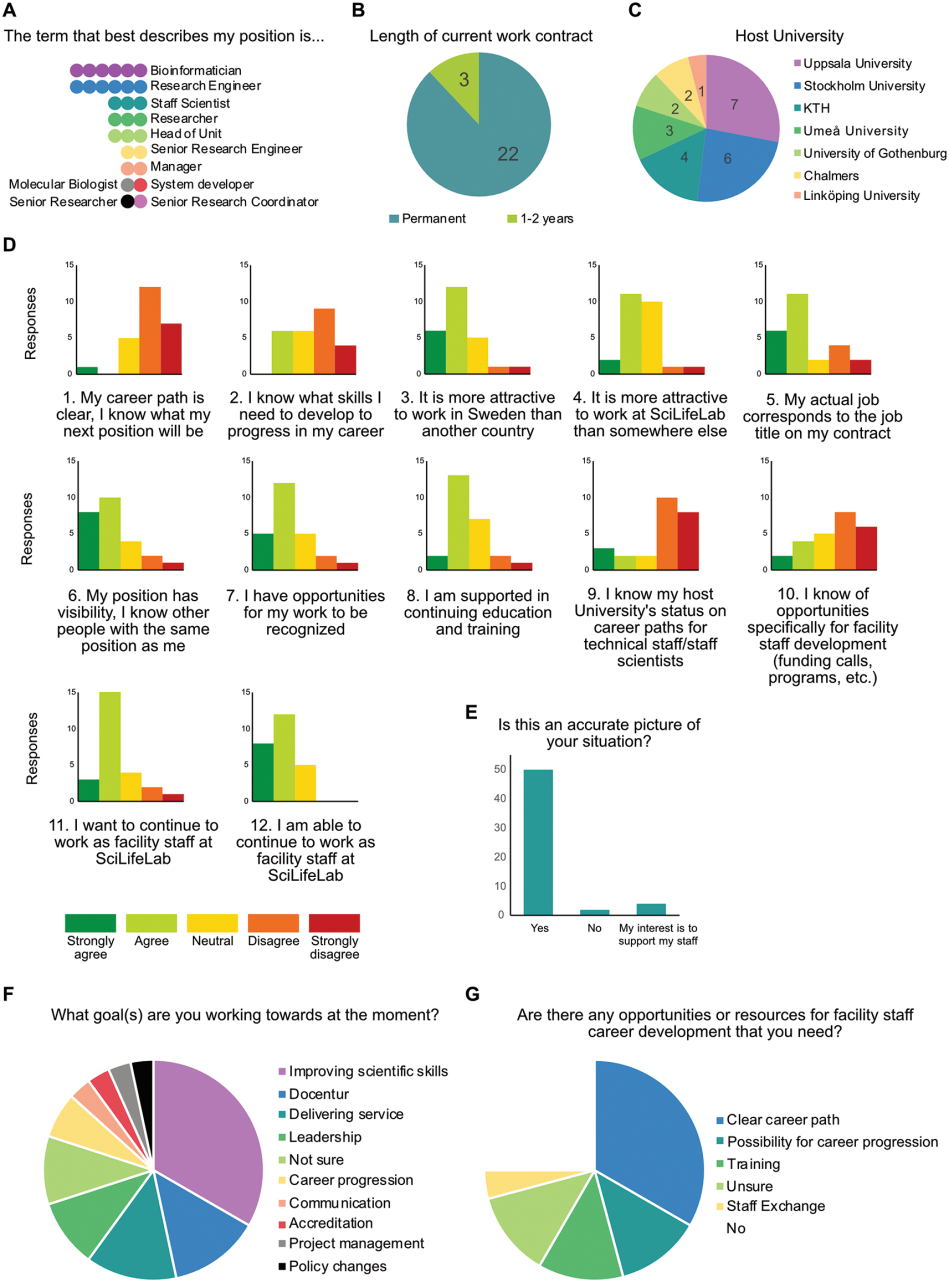
Pre-workshop survey results. Responses to the online survey compiled by question. Number of participants who responded was 25 out of a possible 65 (40% response rate). A-D) Distribution of responses to pre-workshop survey questions on demographics and views on career paths in Sweden. E) Menti response during in-person workshop (56 responses). This question was posed following presentation of results A-D to the participants. Ten responses of ‘Yes’ indicated they had also responded to the pre-workshop survey. F-G) Distribution of responses to pre-workshop survey questions on future development and resourcing.

The pre-workshop survey was designed to understand the community demographics and current views on scientific career paths available to research infrastructure and core facility staff across universities in Sweden. To do this, questions were formulated with input of the 2019 survey by Stefan Nordlund, ^9^ surveys of early career researchers in Sweden, ^12^ global survey of core facility staff needs, ^4^ as well as the pillars of the Technician Commitment. ^5^ Of 27 responses received prior to the workshop (two respondents held more than one job title), the most common positions reported were Bioinformatician and Staff Scientist (including variations of Research Engineer, Researcher, and Senior Research Engineer, Figure 1A). Of the respondents, 88% held a permanent work contract, with the other 12% holding employment contracts of 1-2 years ( Figure 1B). This distribution is similar to what has been reported for European staff scientists in other surveys. ^4^ While seven Swedish universities were represented in the survey data, the participants were heavily weighted towards Uppsala University and Stockholm University ( Figure 1C). Participants from Stockholm-Uppsala area universities made up 68% of pre-workshop survey participants, consistent with 67% of all registered participants from a total of 11 institutions.

A series of twelve statements on career progression were posed in the survey, and responses grouped generally into clear agreement or disagreement with the statement ( Figure 1D). Generally positive responses indicated that Sweden and SciLifeLab are attractive places to work (Q3, Q4), that positions are stable, recognized, and visible (Q6, Q7, Q11, Q12), and that continuing education and training are supported (Q8). Generally negative responses indicated that both the career paths and metrics required for progression in a non-academic track career in Sweden are unclear (Q1, Q2, Q9), and that information is lacking on opportunities designed for the experts in these careers (Q10). Collectively, this suggests that a non-academic track position at a university in Sweden is attractive and stable but is associated with uncertainty in career progression. Finally, when the larger group of in-person workshop participants were asked if this picture was accurate to their situation, the majority of responses agreed (50/56, 89%, Figure 1E).

While staff scientists reported a lack of knowledge on or possibility for career progression, this was not associated with a lack of intrinsic goals or motivation, or a lack of support in continuing education and training. Survey respondents were asked what goals they were actively working towards and a variety of targets were reported ( Figure 1F), with the largest number of responses being improving technical skills (33%). While many responses indicated uncertainty over career development, only 10% of responses indicated uncertainty over goals. When asked if there were opportunities or resources staff scientists needed for career development, 46% of responses indicated the need for a clear career path or the possibility for career progression ( Figure 1G), compared with 17% responses indicating a need for further training or mentorship, and 38% responses of not needing or being unsure if further resources were needed. These results illustrate facility staff as a motivated group that are facing a situation where meeting goals and advancing skills are not necessarily associated with career progression.

### Workshop presentation of survey results and career paths progress 2019-2024

**Figure 2.**
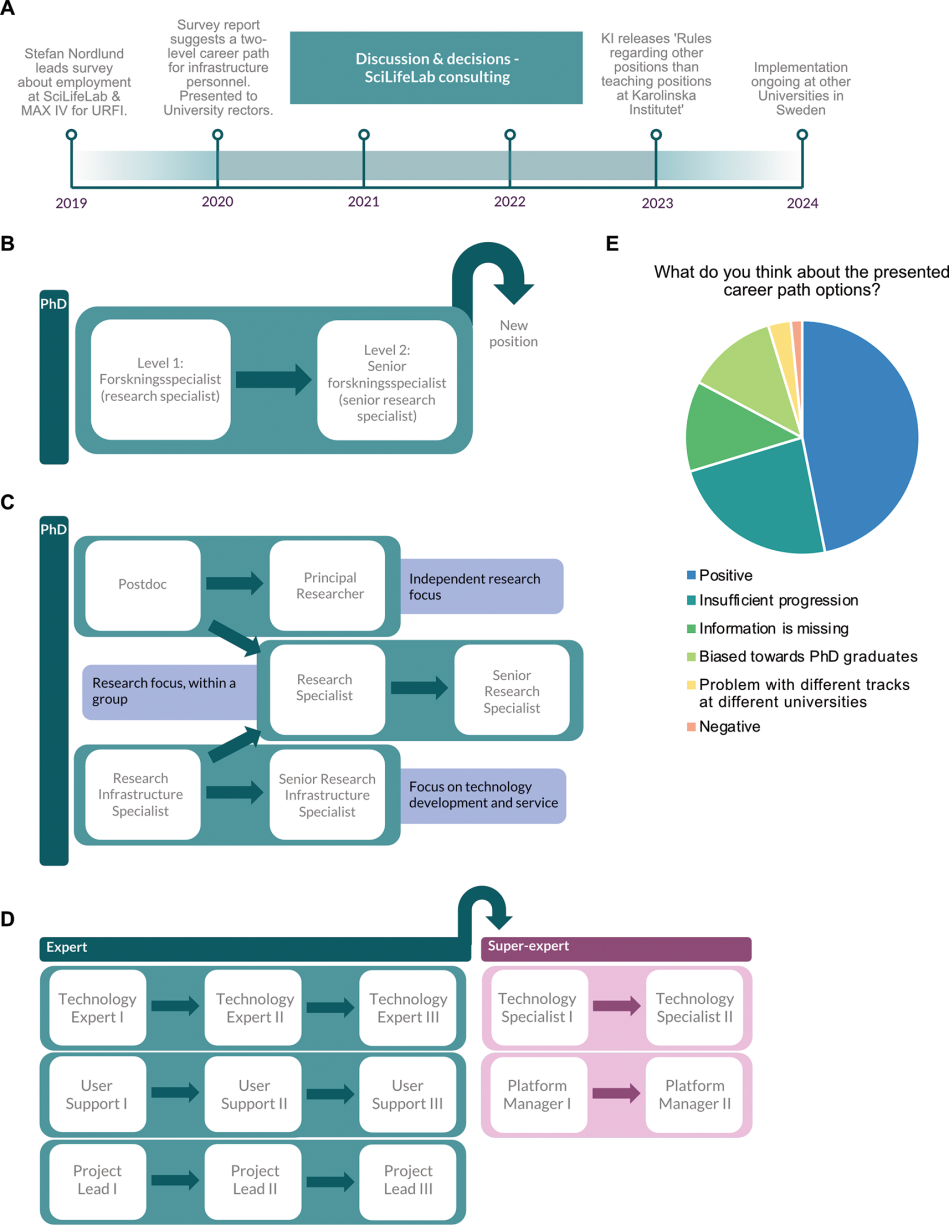
Presentation of timeline of career path development in Sweden. A) Timeline of career path development in Sweden, starting from URFI survey. B) Diagram of the two-step career path suggested in the URFI report. ^9^ C) Diagram of the branched career path implemented by the Karolinska Institute. ^10^ D) Diagram of a potential branched career path for Universities in Sweden. E) In-person response to the proposed career paths (64 responses from 55 participants).

During the in-person Facility Forum workshop, a summary of career path development at Swedish universities was presented to the participants and later made freely available. A timeline of career path development for technical staff was presented to participants ( Figure 2A), beginning with Stefan Nordlund’s recommendation of a two-step career path for staff scientists at Swedish universities ( Figure 2B). The career path structure approved by KI in 2023 ^10^ was also presented ( Figure 2C), as well as more branched paths that have been suggested for universities in Sweden based on work in the UK, including the Technical Specialists Promotional Pathway Pilot at the University of Warwick. ^13^ Following this presentation, the workshop participants were polled for feedback on these career paths. Responses were split between positive and negative ( Figure 2E). While 47% of responses were positive, indicating the view that non-academic track career path development in Sweden is progressing, 53% expressed concerns about insufficient progression in compensation, concerns of how career paths are to be implemented over existing staff structures, and questions of equity in national institutions employing staff from universities with different career path structures (including SciLifeLab). Importantly, 12.5% of responses noted that career paths in Sweden have only so far been addressed for PhD graduates, and that staff scientists holding other degrees are currently excluded from the formal career development process.

### Feedback on career progression from workshop participants

**Figure 3.**
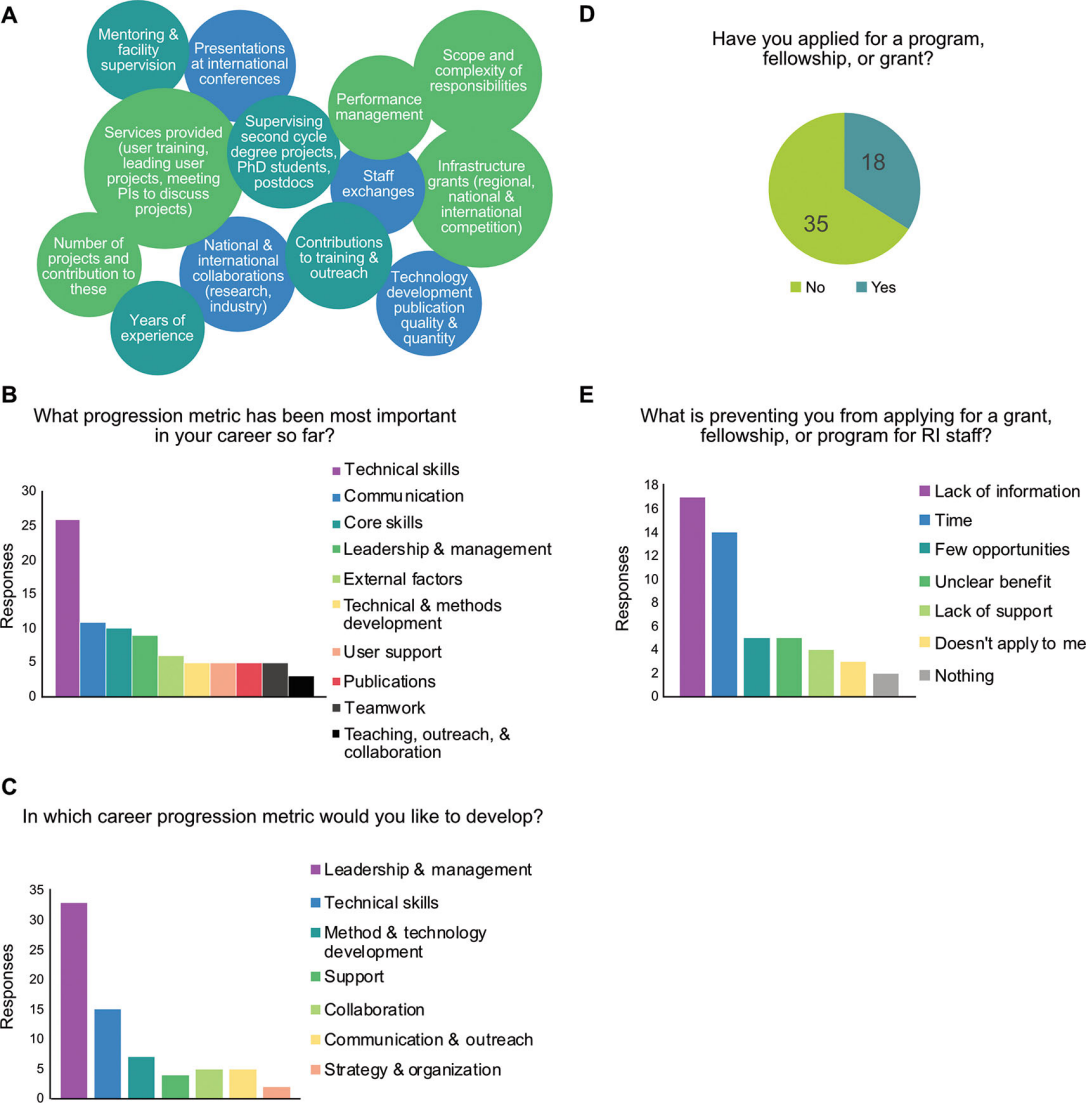
Current career progression needs. A) Presented diagram of possible career progression metrics for infrastructure staff. B) In-person Mentimeter, 64 responses from 43 participants. C) In-person Mentimeter, 85 responses from 47 participants. D) In-person Mentimeter, responses from 53 participants. E) In-person Mentimeter, 50 responses from 42 participants.

As most Swedish universities plan to integrate career paths for staff scientists, a discussion was held to identify the skills, knowledge, and competencies essential for career development beyond the academic tenure track ( Figure 3A). When asked which skills were most important in the development of staff scientists’ careers, technical skills were ranked most highly, followed by communication, core skills (social and character skills), and management abilities ( Figure 3B). Publications was a relatively rare response, although publication quantity and quality are frequently used as indicators. Teaching and collaboration were also relatively rare responses, indicating a need to recognize these efforts and incorporate them into career structures in order to support organizational growth, as planned for at SciLifeLab through agreements such as CoARA. ^8^ When polled about which career progression metrics the workshop participants would like to develop in, the primary response was leadership & management skills ( Figure 3C), suggesting the development of a management strand ^13^ may be beneficial to diversifying career progression structures.

Finally, when asked if they had applied for a program, fellowship, or grant available specifically for research infrastructure staff, 66% of workshop participants responded that they had not ( Figure 3D). The primary barriers to pursuing professional growth for scientific staff were insufficient information and limited time ( Figure 3E), while several responses highlighted a scarcity of opportunities, some noting that applying for them provided little benefit without a well-defined career path structure.

## Discussion

In order to support and retain expertise in Swedish core facilities and research infrastructures, clear career development strategies must be implemented to foster growth at the individual, group, and institutional level. Stefan Nordlund’s survey indicated that 38% of respondents had held their position for over 5 years at a SciLifeLab unit, ^9^ and the view of a SciLifeLab position as stable and attractive ( Figure 1D) supports longevity of technical staff despite the current lack of horizontal and vertical career progression structure that can develop and utilize this expertise. During the course of this workshop, a comment was shared indicating that an employee’s career advancement would only be possible if their supervisor vacated their position. Furthermore, as a presentation of SciLifeLab demographics from the Facility Forum reported that one third of SciLifeLab staff do not hold a PhD, career development has only begun to be addressed for two thirds of infrastructure staff. This clearly underscores the ongoing need to diversify career tracks for facility staff, fostering organizational flow and supporting the development of the highly skilled professionals who drive research facilities.

A notable aspect of this workshop was the recurring feedback highlighting a lack of information ( Figure 1D, Figure 1G, Figure 2E, Figure 3E). During the workshop, participants shared insights on the career path status at certain universities, though the status remained unknown for others. While the workshop facilitated some knowledge transfer, staff scientists in Sweden consistently reported being disconnected from information regarding the plans for their career development. Increasing efforts to gather and share existing resources and information with research infrastructure staff in Sweden would be helpful in ameliorating this issue.

Lastly, less than half of workshop participants had applied for a program, fellowship, or grant ( Figure 3D), suggesting that efforts to cultivate, recognize, and foster excellence in expert technical staff could benefit from increased communication with staff scientists. Expert technical staff have many years of educational investment in their technical capacity, and recognition of this expertise as an important resource has been prioritized within Europe (see funding for initiatives such as ARISE, RItrain, ELITMa, eLEAD, and InnoCore) and Sweden (initiatives such as the ITM program from the Swedish Foundation for Strategic Research). ^11^ Initiatives like SciLifeLab’s ongoing work within CoARA also aim to promote broader recognition of research contributions in Sweden. However, fostering national support for the development and acknowledgement of diverse research infrastructure staff roles is essential to highlighting the vital contributions of staff scientists to scientific discovery.

### Preregistered data analysis

This study was not preregistered at an independent registry.

## Ethical considerations

According to the Swedish Ethical Review Act (Lag (2003:460) om etikprövning av forskning som avser människor), this study does not contain methods or information requiring ethical review or approval. Anonymization of survey data from the workshop has been ensured, and we confirm that this anonymization of survey data has not distorted scientific meaning.

## Author contributions

KS participated in conceptualization, data curation, formal analysis, investigation, methodology, visualization, and writing of original draft as well as review and editing. JFR, AJJ, JLG, SS and CS participated in conceptualization, project administration, methodology, supervision, and writing review and editing. JML participated in conceptualization, project administration, supervision, and writing of the original draft as well as review and editing.

## Data Availability

Raw survey data, which includes long-form answers and identifying information, cannot be made openly available in adherence with EU data protection laws. Survey questions and the anonymized data used to create the figures is available as follows: ^14^ SciLifeLab Data Repository. Recruitment to Retirement Workshop 2024, http://doi.org/10.17044/scilifelab.28661375. This project contains the following underlying data: Recruitment to Retirement Survey Questions. SurveyQuestions.docx Recruitment to Retirement Anonymized Figure Data. SurveyData.xlsx Data are available under the terms of the Creative Commons Attribution 4.0 International license (CC-BY 4.0).
