# Supplementary material for: From Recruitment to Retirement: Research Infrastructure Staff Views on the Diversification of Scientific Career Paths at Universities in Sweden in 2024
Source: F1000Res. 2025 Jul 4;14:652. [Version 1] doi: 10.12688/f1000research.164794.1 (PMC12402762; doi:10.12688/f1000research.164794.1)
Supplement: Peer review history

# Reviewer report: Reviewer response for version 1

**DOI:** 10.5256/f1000research.181367.r401511

**Published:** 2025-09-01

**Authors:** Fran Bagenal

**Affiliations:** 1 Laboratory for Atmospheric and Space Physics, University of Colorado, Boulder, Colorado, USA

The report presents a study of career paths in the life sciences in Sweden via a survey of attendees from the SciLifeLab to their semi-annual Facility Forum. The survey involved only the 65 attendees of the forum with a limited (40%) response rate. The authors present their questions and results clearly and provided a sense that more career advice would be useful for the employees. The authors covered a range of career issues and obtained a sense of where the system was working well - and sometimes not so well. Their conclusions confirmed a general trend that I have observed across multiple nations that standard academic tracks in the sciences - particularly conventional sciences such as physics, chemistry, biology - follow a well-defined, narrow path. But career paths in the applied sciences or new research fields are not so well defined and can involve a braided path with multiple potential options, junctions, and destinations.

 The authors note that the study provides some useful guidance to management of the relevant institutions. It would have been nice to see some more specific suggestions of actions that should be taken  at different levels: A: individual; B: institutional; C: national, say via a professional organization. 

 But to make major progress the study needs to be followed up with a much more expansive survey that 1: involves significantly larger numbers of (e.g. carried out by a professional organization), who are all encouraged to respond by their institution to obtain a high response rate; and 2: includes basic demographics questions such as age, gender, ethnicity, education, whether first-generation at college, etc., in addition to the career questions in this study. Such demographics data will allow quantitative assessments of what factors might be related to career issues. For example, do people from underrepresented populations and/or first-generation college attendees require more direct information and/or guidance along their career path? Do people with a traditional cultural background and/or well-educated parents tend to assume a traditional academic career path? The demographics information will also allow the profession to compare between various types of careers in different science fields. Comparison of demographics statistics for difference science fields internationally can also be revealing as well as perhaps lead to examples of what actions can be effective at different levels (A, B, C above).

Is the work clearly and accurately presented and does it cite the current literature?

Partly

If applicable, is the statistical analysis and its interpretation appropriate?

I cannot comment. A qualified statistician is required.

Are all the source data underlying the results available to ensure full reproducibility?

Yes

Is the study design appropriate and is the work technically sound?

Partly

Are the conclusions drawn adequately supported by the results?

Yes

Are sufficient details of methods and analysis provided to allow replication by others?

Partly

Reviewer Expertise:

Space environments of planets and moons of the outer solar system. Over the past ~20 years I have also worked with the American Institute of Physics in carrying out demographics studies of the space and planetary science professions and participated in workforce/climate studies.

I confirm that I have read this submission and believe that I have an appropriate level of expertise to confirm that it is of an acceptable scientific standard.

# Reviewer report: Reviewer response for version 1

**DOI:** 10.5256/f1000research.181367.r398574

**Published:** 2025-08-23

**Authors:** Emma Karoune

**Affiliations:** 1 The Alan Turing Institute, London, UK

1. Is the work clearly and accurately presented and does it cite the current literature?

Partly – The paper is clearly written and sets out the aim to collect current views on how scientific career path diversity has developed over the last four years in Sweden. And it is also clearly set out with sections for methods, results and discussion, but in some sections more detail is needed to fully understand how the study has been conducted and more discussion bringing in work from the wider European context would improve the paper greatly.

 The introduction briefly describes the current context around the project in Sweden and Europe more broadly. They could add several other relevant references:

- This reference includes research infrastructure professionals:
  - White, A., & Green, D. (2025). Minimum Viable Skillsets: a Briefing for Competence Centres. Zenodo. https://doi.org/10.5281/zenodo.15025706
- A reference for the Research Software Engineering role in addition to reference 7, for example:
  - J. Cohen, D. S. Katz, M. Barker, N. Chue Hong, R. Haines and C. Jay, "The Four Pillars of Research Software Engineering," in IEEE Software, vol. 38, no. 1, pp. 97-105, Jan.-Feb. 2021, doi: 10.1109/MS.2020.2973362.
- Policy briefing:
  - Karoune, E., & Sharan, M. (2025). Policy briefing - Diversifying Professional Roles in Data Science. Zenodo. https://doi.org/10.5281/zenodo.14946850

 Although there is a conclusion in the abstract, there is no conclusion section in the main paper – this should be added.

 Some figures could be made clearer:

- In most figures, the colour is used for the key. I don’t think the colour palette used for the graphs is one that is colour blindness friendly – please could this be changed to allow greater accessibility.
- Figure 1G – the ‘No’ needs a different colour or a boarder to make it clearer.
- Figure 3A – add a title to keep consistent with graphs.

2. Is the study design appropriate and the work technically sound?

 Partly – I’m not sure that the study design meets the aim of the paper due to under-representative and small sample size – only sampling one RI, small sample size of survey and workshop - and the lack of detail given in the method and analysis making it hard to understand decisions made in the study.

 These issues could be easily addressed without too much effort from authors to make the paper much more robust.

 The aim of the paper is a national focus on Sweden in terms of changes in career pathways. But the paper only collects data from one RI out of 60 in Sweden. This does not seem a representative sample for the whole country and the authors also point out that there is a bias in the results towards two universities making the data even less representative. I suggest that the aim of the paper is slightly altered to reflect this, such as being a survey of staff at one RI to understand changes at organisational level and reflections on these results at a national level.

 Small sample size - the pre-workshop survey was only answered by 25 people out of the 65 people signed up to the workshop. 65 people was the maximum for the workshop, but this is 65 out of 600 people in SciLifeLab. It would have been better to send the survey out to all staff to allow a greater number of people to respond and therefore this would have been more representative of the organisation as a whole. The authors need to discuss the limitations of the results more explicitly, such as stating that the results may be biased due to small sample size and many respondents coming from two universities.

 3. Are sufficient details of methods and analysis provided to allow replication by others?

 No – Much more detail needs to be included for the methods and analysis. With this detail, someone else should be able to replicate your method and analysis even though you are not able to provide raw data – this is not possible at the moment.

 The method should be more detailed. There are details in the README file that should be also written in the paper, such as details of how the survey data was collected/administered, how raw data was changed/processed, what software was used for data processing and making the figures?

 There is also a lack of detail about the analysis – How many people coded the data and who were they? How were the bins/themes decided on? Was saturation reached? How was this determined?

 It needs to be made clearer that most questions in the survey were open response but the graphs presented show the themed answers. At the moment, you can only really understand this if you go to look at the data and survey questions linked at the end of the paper.

 5. Are all the source data underlying the results available to ensure full reproducibility?

 No – there seems to be some restriction on the availability of the raw data – this should be checked again. Fully anonymised raw data could be shared openly, even long-form answers as they could be redacted to ensure anonymity, but this does depend on the consent that has been given by respondents.

 6. Are the conclusions drawn adequately supported by the results?

 The conclusions in the abstract are supported by the results but there could be more discussion generally and addition of a conclusion section in the main text.

 Figure 2E – it is reported that 53% were negative about new career paths. This is a significant finding, could you point out in more detail what you think is missing? and so what could be done about this? Further national consultation? The authors have pointed out career paths don’t currently include staff that don’t hold PhDs – how could this be rectified? How has this been solved elsewhere? Could it be that number of years work experience is considered equivalent to PhD for technical roles?

 The authors point out lack of information and knowledge transfer as an issue and they say that this could be tackled by increasing efforts to gather but how? – creating a community, society, association? How has this been done elsewhere? How supported at national level? It could also be suggested that technical staff in Sweden need to be part of European wide initiatives such as Skills 4 EOSC, and ELIXIR focus groups, to align with wider efforts in this area.

 Point in discussion around lack of applications for programs, grants, etc – again the authors should suggest how this could be overcome by looking elsewhere – in UK, UKRI now allow Research Technical Professionals to lead grants and are funding multiple networks to enable communities of practice for different technical roles ( https://www.ukri.org/news/new-funding-to-support-research-technical-professionals/ and https://www.ukri.org/opportunity/ukri-digital-research-technical-professional-skills-networkplus/).

 Authors could also suggest that a larger and/or longitudinal survey/study is needed to truly gather national and long-term understanding of this issue.

Is the work clearly and accurately presented and does it cite the current literature?

Partly

If applicable, is the statistical analysis and its interpretation appropriate?

Not applicable

Are all the source data underlying the results available to ensure full reproducibility?

No

Is the study design appropriate and is the work technically sound?

Partly

Are the conclusions drawn adequately supported by the results?

Yes

Are sufficient details of methods and analysis provided to allow replication by others?

No

Reviewer Expertise:

Broad experience in Data Science infrastructure and qualitative research. Specific research experience related to data science skills and roles.

I confirm that I have read this submission and believe that I have an appropriate level of expertise to confirm that it is of an acceptable scientific standard, however I have significant reservations, as outlined above.

# Reviewer report: Reviewer response for version 1

**DOI:** 10.5256/f1000research.181367.r398572

**Published:** 2025-08-21

**Authors:** Julián D. Cortés

**Affiliations:** 1 Universidad del Rosario, Bogotá, Colombia

Introduction

 Consider broadening the context of your research within the larger peer-reviewed literature on scientific (i.e., research performing) careers. For instance, you mention the CoARA framework as a benchmark for acknowledging career diversity. Just by using this criterion for an exploratory document search in titles and abstracts, over 20 works can be traced in OpenAlex. Here's a query to replicate this: https://openalex.org/works?page=1&filter=title_and_abstract.search:%22Coalition+for+Advancing+Research+Assessment%22

 It's unclear whether the aim, purpose, and research questions (RQs) of this paper align with those of the workshop, which was "to ask infrastructure staff for their current views on how scientific career path diversity has developed over the past four years in Sweden." If the paper's goal is to report the workshop results, consider extending the aim's description by adding SMART (Specific, Measurable, Achievable, Relevant, and Time-bound) criteria to define your objectives. Alternatively, if you're using RQs, consider employing the PICO (Population, Intervention, Comparison, and Outcomes) or SPICE (Setting, Perspective, Intervention, Comparison, Evaluation) standards.

 Methods:

 It would help the reader to visualize your research design before presenting the results. A diagram or figure illustrating the design would be beneficial. Furthermore, while you state that "free text entries were coded to remove identifying information and shown in aggregate," and "Realtime responses were coded to remove identifying information and are presented alongside the survey results," you don't elaborate on the specific qualitative analytical techniques used (e.g., whether thematic analysis, content analysis, or another systematic approach was applied to categorize and interpret these qualitative data).

 A more detailed explanation of how themes or patterns were identified from these open-ended responses would significantly enhance the methodological rigor and transparency of your study. Also, describing how codes were developed (inductively, deductively, or both), how disagreements in coding were resolved, and how themes and subthemes emerged would offer greater transparency and confirmability.

 Could it be of interest controlling for socio-demographic variables? (e.g., gender, current lifecycle, migration status, etc.) This would add considerably more scope and significance to a wider discussion on research careers.

 Results:

 There's a heavy weighting of participants from the Stockholm-Uppsala area, which is consistent with the overall registered participants from 11 institutions. While this is acknowledged, a more explicit discussion of how this geographical concentration might influence the generalizability of your findings is needed.

 Additionally, if participants from 11 institutions out of 40 facility units participated in the workshop (more information is needed to understand the correspondence between units/institutions), consider extending the justification and limitations of this sample of institutions/units/participants regarding the study's significance.

 Avoid using pie charts. In general, your visualizations are great. I'd simply suggest further good practices of data visualization, such as those outlined here: [Reference 1] https://journals.sagepub.com/doi/10.1177/15291006211051956

 Discussion:

 Enrich the discussion of your study by incorporating the improved literature review you suggested earlier. Extend the limitations and also the next stages of the project. For instance, with this type of data, authors could be interested in using another methodologies such as grounded theory or case study? Incorporating other data collection techniques such as in-depth semi-structured or unstructured interviews or thematic analysis?

Is the work clearly and accurately presented and does it cite the current literature?

Partly

If applicable, is the statistical analysis and its interpretation appropriate?

Partly

Are all the source data underlying the results available to ensure full reproducibility?

Partly

Is the study design appropriate and is the work technically sound?

Partly

Are the conclusions drawn adequately supported by the results?

Partly

Are sufficient details of methods and analysis provided to allow replication by others?

Partly

Reviewer Expertise:

Science Policy; Research Evaluation; Scientometrics

I confirm that I have read this submission and believe that I have an appropriate level of expertise to confirm that it is of an acceptable scientific standard, however I have significant reservations, as outlined above.

## References

[1] : The Science of Visual Data Communication: What Works. *Psychological Science in the Public Interest* .2021;22(3) : 10.1177/15291006211051956 110-161 10.1177/15291006211051956 34907835

# Reviewer report: Reviewer response for version 1

**DOI:** 10.5256/f1000research.181367.r398575

**Published:** 2025-08-21

**Authors:** Stefano Gaburro

**Affiliations:** 1 Independent Researcher, Deutschland, Germany

The work by Schroeder et al. addresses the pressing issue of career path diversification for research infrastructure staff at Swedish universities. This work is both timely and relevant, as the professional trajectories of staff scientists, engineers, and technical specialists are gaining attention internationally but remain underdeveloped in many national contexts. The authors employ a mixed-methods approach, integrating both survey data and a facilitated workshop, which enables a broad and nuanced understanding of staff perspectives across SciLifeLab-affiliated institutions. The manuscript is clearly structured, the presentation of data is effective, and the discussion thoughtfully connects empirical findings with national and international policy debates.

 On the less positive side, the study is constrained by a relatively small and non-representative sample. The majority of survey and workshop participants are drawn from just two institutions, and the voluntary nature of participation introduces a risk of selection bias, with perhaps only the most engaged or concerned staff opting to respond. Methodological details, particularly regarding qualitative data analysis, could be strengthened, and the study's limitations, especially those concerning generalizability, would benefit from a more comprehensive discussion. Moreover, the lack of inferential statistics and limited subgroup analysis may prevent some important patterns from emerging. Finally, while the literature review is thorough regarding European initiatives, the discussion could be further enhanced by integrating more international perspectives.

 The detailed review below provides actionable recommendations to address these limitations and further develop the manuscript’s strengths:

 1. Methods Section (Suggested Rewrite)

 The workshop was conducted during the 2024 SciLifeLab Facility Forum, a biannual event open to all infrastructure staff across SciLifeLab’s platforms and units. Participation in the workshop was voluntary, with a cap of 65 attendees to ensure manageability. The study utilized a three-phase approach: (1) a pre-workshop survey distributed by email to all registered participants; (2) an in-person workshop presentation of preliminary findings; and (3) real-time collection of participant feedback using Mentimeter (Mentimeter AB, Sweden), supplemented by small group discussions. Survey questions were developed based on prior published instruments [refs 4, 9, 12], reviewed by the project team for clarity, and piloted internally. Free-text survey responses and workshop comments were anonymized, coded independently by two authors using thematic analysis, and aggregated for presentation. Any discrepancies in coding were resolved through discussion.

 2. Limitations Section (Expanded)

 This study is subject to several important limitations. First, the sample size is modest, with a 40% response rate to the pre-workshop survey and overrepresentation from Uppsala and Stockholm Universities. This limits the generalizability of our findings to all Swedish research infrastructure staff. Second, self-selection bias may have occurred, as individuals more interested in career path issues may have been more likely to participate. Third, while responses were anonymized, the risk of deductive disclosure remains for small or unique subgroups. Finally, our analysis relied primarily on descriptive statistics; the absence of inferential statistical tests limits the ability to draw firm conclusions about subgroup differences. Future studies should aim for broader sampling, stratified analysis, and, where possible, inclusion of inferential methods to strengthen the evidence base.

 3. Results: Data Analysis and Table Suggestions

 Responses to the twelve career progression statements are summarized in Table 1, stratified by contract type and institution. While most respondents agreed that Sweden and SciLifeLab are attractive places to work, a substantial minority expressed uncertainty regarding the availability of career paths and progression metrics.

 Statement

 % Agree (Permanent)

 % Agree (Fixed-term)

 % Agree (Stockholm-Uppsala)

 % Agree (Other)

 Sweden/SciLifeLab attractive

 90%

 80%

 92%

 85%

 Career paths are clear

 35%

 10%

 30%

 25%

 *(Example data—replace with actual figures as appropriate.)*

 4. Figures and Referencing

 Revise figure references for clarity. For example:

 "As shown in Figure 1F, improving technical skills was the most commonly reported goal, accounting for 33% of responses."

 Ensure all figure panels are clearly identified (A, B, C...) in both captions and the main text. Consider adding one summary table (e.g., 'Barriers to Career Progression: Survey vs. Workshop Responses') to complement the figures.

 5. Policy Implications and International Context

 These findings have significant implications for policy and institutional practice. The lack of clear, equitable career paths for research infrastructure staff mirrors challenges observed in other European countries and internationally [refs 1, 4, 7]. To address these issues, Swedish universities and funding agencies should consider adopting transparent, multi-track career frameworks and invest in targeted communication and mentorship initiatives. Lessons may be drawn from the UK's Technician Commitment and the Netherlands' national data stewardship programs, which have improved recognition and progression opportunities for staff scientists. Broader international benchmarking and harmonization of career structures may further enhance the competitiveness and sustainability of Swedish research infrastructure.

 6. Literature Review: Additional References

 Add references to recent US and Asia-Pacific initiatives on staff scientist career development (e.g., NIH Staff Scientist policies, Australia’s EMBL core facility career frameworks).

 7. Ethical Considerations (Expansion)

 All data were stored securely and access was restricted to the study team. To further minimize re-identification risks, demographic subgroup data are reported only in aggregate. The study was conducted in accordance with the principles of the Declaration of Helsinki.

Is the work clearly and accurately presented and does it cite the current literature?

Partly

If applicable, is the statistical analysis and its interpretation appropriate?

Partly

Are all the source data underlying the results available to ensure full reproducibility?

Partly

Is the study design appropriate and is the work technically sound?

Partly

Are the conclusions drawn adequately supported by the results?

Yes

Are sufficient details of methods and analysis provided to allow replication by others?

Partly

Reviewer Expertise:

Neuroscience and Academic Career Development

I confirm that I have read this submission and believe that I have an appropriate level of expertise to confirm that it is of an acceptable scientific standard, however I have significant reservations, as outlined above.
